# PD-L1 expression in bladder cancer and metastasis and its influence on oncologic outcome after cystectomy

**DOI:** 10.18632/oncotarget.19913

**Published:** 2017-08-03

**Authors:** Renate Pichler, Isabel Heidegger, Josef Fritz, Melanie Danzl, Susanne Sprung, Bettina Zelger, Andrea Brunner, Andreas Pircher

**Affiliations:** ^1^ Department of Urology, Medical University Innsbruck, Innsbruck, Austria; ^2^ Department of Medical Statistics, Informatics and Health Economics, Medical University Innsbruck, Innsbruck, Austria; ^3^ Department of Pathology, Division of General Pathology, Medical University Innsbruck, Innsbruck, Austria; ^4^ Department of Internal Medicine V, Hematology and Oncology, Medical University Innsbruck, Innsbruck, Austria

**Keywords:** bladder cancer, cystectomy, survival, PD-L1, immunotherapy

## Abstract

Platinum-based chemotherapy is the standard of care in metastatic bladder cancer. With the approval of various checkpoint inhibitors, immunotherapy has revolutionized the traditional treatment modalities. The aim of the study was to evaluate whether PD-L1 expression on tumor cells (TCs) and tumor-infiltrating immune cells (ICs) can be used as biomarker to predict recurrence-free survival (RFS), overall survival (OS) and disease-specific survival (DSS) in bladder cancer patients after radical cystectomy (RC) developing disease recurrence followed by first-line chemotherapy. PD-L1 was measured on formalin-fixed, paraffin-embedded tissue sections of RC specimens in all patients (n=61) and in 27 matched metastatic biopsy samples by immunohistochemistry. PD-L1 expression on TCs was defined by the percentage of PD-L1 positive tumor cells (< 1%= IC0, ≥1% but <5%=IC1, ≥5 %=IC2/3), and was considered negative or positive for ICs. On 27 paired samples, IC1/2/3 score on TCs was homogeneous distributed with 59.3% in primary tumors and metastases, but with a high discordance rate of 44.4% of PD-L1 positivity on ICs. High PD-L1 expression (IC2/3) on TCs was more frequently seen in histologic subtypes of urothelial cancer compared to pure urothelial cancers (46.2% *vs*. 20.8%; *p*=0.002). PD-L1 expression on TCs in primary tumors (IC2/3 *vs*. IC0, median: 3.2 *vs*. 13.8 months, *p*=0.019) and metastatic sites (IC2/3 *vs*. IC0, median: 6.1 *vs*. 21.8 months, *p*=0.014) was associated with poor chemo-response, represented by significant shortened DSS. These results suggest that PD-L1 may be a potential target being involved in chemo-resistance mechanisms and poses potential for therapy stratification in the future.

## INTRODUCTION

Bladder cancer is a highly immunogenic malignancy, including T cell-inflamed and non-T cell-inflamed tumors [1]. Despite the successful introduction of various checkpoint inhibitors in bladder cancer, the objective response rate (ORR) is limited, and lies within 46.4% ORR in programed death –ligand 1 (PD-L1) positive [2] and between 0% and 26.2% ORR in PD-L1 negative patients [2–3]. Hereby the quantification of overall PD-L1 expression on tumor cells or tumor-infiltrating immune cells ranged in primary tumor between 32% and 66% [2–3]. Thus, most patients do not benefit from immunotherapy and therefore the characterization of resistance mechanisms and identification of predictive biomarkers are of utmost clinical importance. Thereby, the tumor microenvironment seems to play an important role for predicting response to Bacillus-Calmette Guérin (BCG) immunotherapy [4–6] in non-muscle invasive bladder cancer (NMIBC), and may also regulate the resistance to checkpoint inhibitors in metastatic bladder cancer disease [7]. Furthermore, immune gene expression profiling identified three molecular pathways (B-catenin overexpression, FGF3 mutations and PPAR-g activation) linked to a non-T cell-inflamed tumor microenvironment, being responsible for intrinsic resistance to immunotherapies [7], Supplementary Figure 1. On the contrary, a T-cell inflamed tumor microenvironment is associated with increased CD8^+^ T cell infiltration (Supplementary Figure 1), higher amount of tumor-infiltrating lymphocyte (TIL) clonality, which correlates with higher PD-L1 expression [8], higher mutation load [9] and consecutive radiographic response to checkpoint inhibitors such as atezolizumab [9], supporting an adaptive immune response-mechanism [8, 10]. Moreover, an increased intratumoral CD8^+^ T cell infiltration was predictive of significantly better disease-free and overall survival (OS) in muscle-invasive bladder cancer (MIBC) [11–12]. In responders to immunotherapy, low T cell receptor (TCR) clonality was noticed in pretreatment blood, whereas TIL clones expanded more rapid and robust during immunotherapy. These findings underline the dynamic changes and complexity of the anti-tumor immune response during treatment with checkpoint inhibitors [13]. In addition, the tumor microenvironment and molecular subtypes in MIBC (luminal, basal, luminal-infiltrated and claudin-low) seem to play also an important role in the response or resistance to chemotherapy [8, 14]. For example, claudin-low tumors, are resistant to cisplatin-based neoadjuvant chemotherapy [14] and represent a molecular subtype of bladder cancer with the highest expression of immune gene signatures, with an up-regulation of chemokines and cytokines from low PPAR-g activity, allowing continuous NFKB activity and consecutive pro-inflammatory milieu, being primed for immunotherapeutic response [15], Supplementary Figure 1. PD-L1 as a possible novel biomarker in predicting oncologic outcome in MIBC after radical cystectomy (RC) and therapeutic response to chemotherapy or checkpoint inhibitors remains a debated issue, with conflicting evidence [12, 16–17] and discordance between PD-L1 expression in primary tumors and metastatic sites [18].

The aim of presented study was to evaluate PD-L1 expression on tumor cells (TC) and TIL in MIBC specimens at RC as well as in metastatic lesions to investigate the influence of PD-L1 expression on relapse after RC, disease-specific survival (DSS) and OS.

## RESULTS

### Patient characteristics

A consecutive series of 61 chemotherapy-naïve patients were investigated, including 48 men (78.7%) and 13 women (21.3%), with a mean (median, range) age of 67.8 (69, 36-84) years who experienced disease recurrence after RC and were then consequently treated with platinum-based, first-line chemotherapy: 33 (54.1%) patients were cisplatin-fit and received gemcitabine/cisplatin and 28 (45.9%) patients underwent gemcitabine/carboplatin chemotherapy. Histopathological factors and characteristics are described in detail in Table 1. In contrast to pure urothelial cancer, histological variants of urothelial cancer including squamous differentiation (n=9), sarcomatoid (n=3) and micropapillary (n=1) features have been confirmed in totally 13 (21.3%) of 61 patients.

**Table 1:** Histopathological and descriptive characteristics of patients with bladder cancer recurrence after radical cystectomy undergoing first line platinum-based chemotherapy

Parameters	n (%)
**Age**, mean ± SD, median (range), *years*	67.8 ± 10.1, 69 (36-84)
**Gender**	
Male	48 (78.7%)
Female	13 (21.3%)
**Tumor stage**	
pT1	6 (9.8%)
pT2a	5 (8.3%)
pT2b	11 (18%)
pT3a	13 (21.3%)
pT3b	11 (18%)
pT4a	15 (24.6%)
**Concomitant CIS**	
No	27 (44.3%)
Yes	34 (55.7%)
**R Positivity**	
No	47 (77%)
Yes	14 (23%)
**LVI**	
No	23 (37.7%)
Yes	33 (62.3%)
**Number of resected LNs at RC**, mean ± SD, median (range)	19.4 ± 10.3, 17 (6-38)
**pN Status**	
pN-	40 (65.6%)
pN+	21 (34.4%)
**Histologic subtype of urothelial cancer**	
Pure urothelial CA	48 (78.8%)
Subtype of urothelial CA	13 (21.2%)
**PD-L1 score on TCs in RC specimens**	
IC0	34 (55.7%)
IC1	11 (18%)
IC2/3	16 (26.3%)
**PD-L1 score on ICs in RC specimens**	
Negative	14 (23%)
Positive	47 (77%)
**PD-L1 score on TCs in metastatic sites**	
IC0	17 (63%)
IC1	5 (18.5%)
IC2/3	5 (18.5%)
**PD-L1 score on ICs in metastatic sites**	
Negative	15 (55.6%)
Positive	12 (44.4%)

CIS=carcinoma *in situ*; LVI=lympho-vascular invasion; LN=lymph node; RC=radical cystectomy; TC=tumor cell; IC=immune cell.

### Investigations of PD-L1 expression in surgical resected urothelial cancer at RC and biopsy-verified metastasis

PD-L1 expression was evaluated on TC as well as on TILs of primary tumors (n=61) and additionally in 27 available biopsies from metastatic sites.

**Figure 1:**
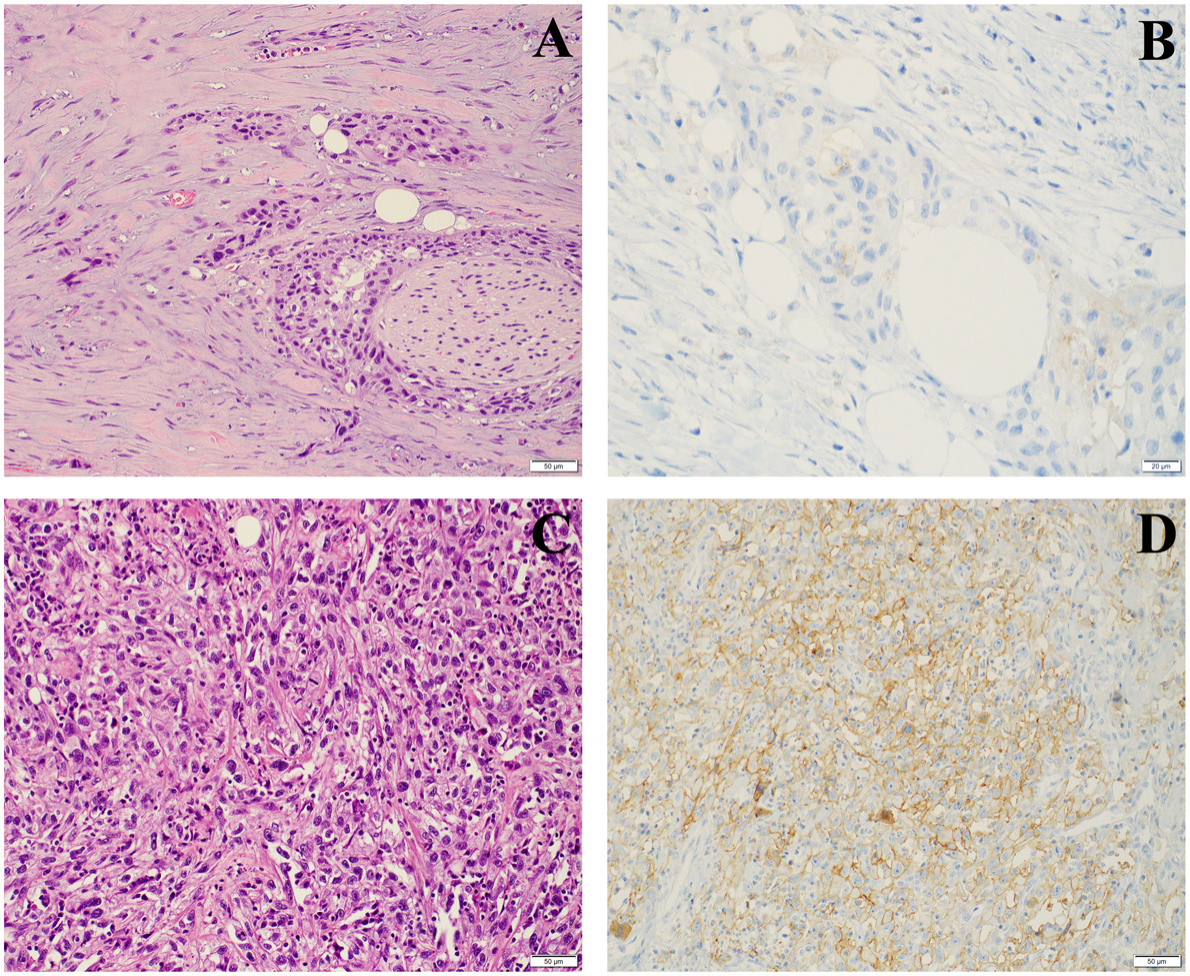
PD-L1 staining on TCs in RC specimens **(A)** Invasive high grade urothelial carcinoma with desmoplastic stroma, low inflammation (H&E) and **(B)** only faintly perceptible (IC0) PD-L1 staining. **(C)** Urothelial carcinoma with sarcomatoid differentiation (H&E) and **(D)** a high score PD-L1 (IC2/3) expression.

**Figure 2:**
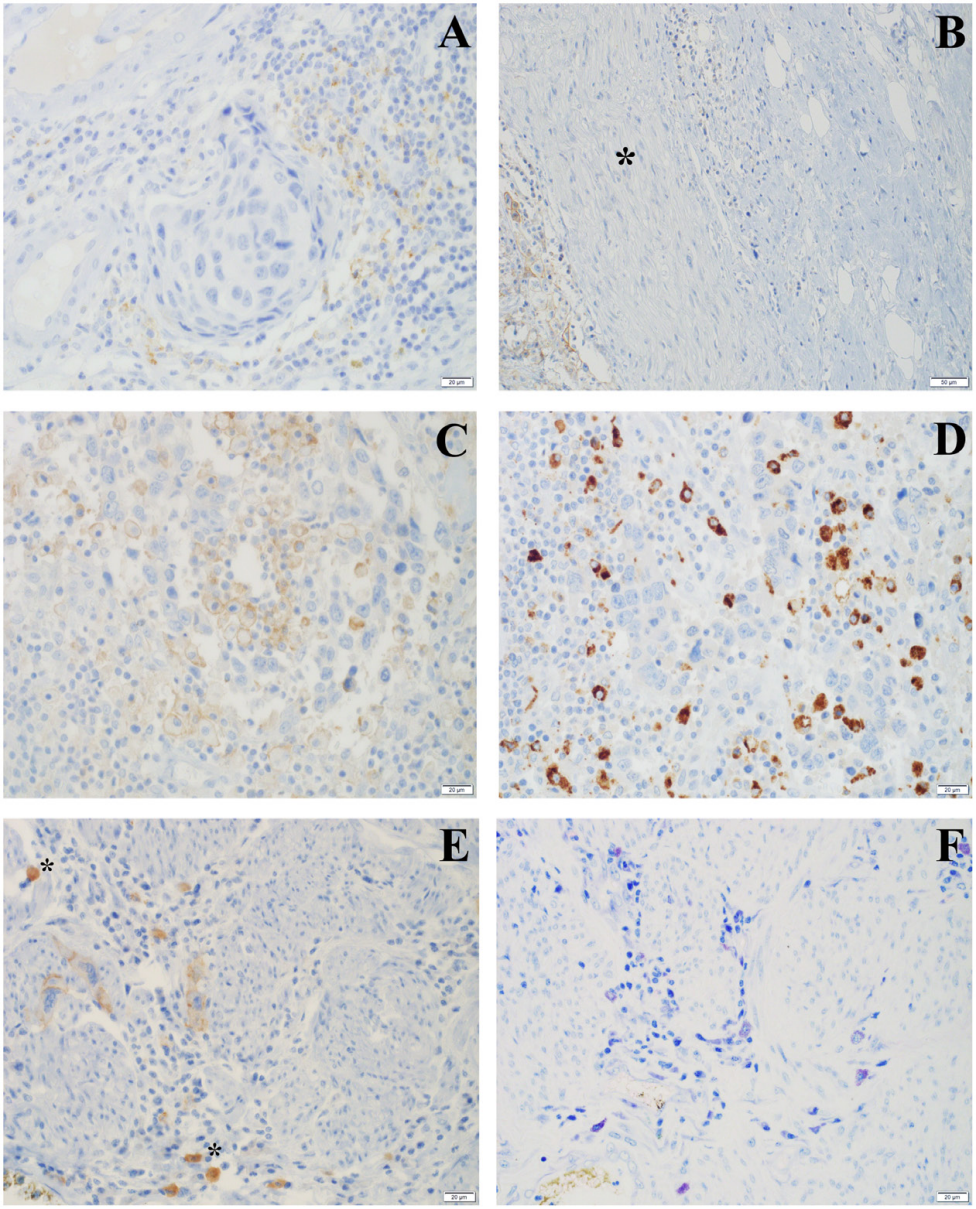
PD-L1 staining on ICs in RC specimens **(A)** Invasive high grade urothelial carcinoma surrounded by a dense infiltrate with partial PD-L1 expression (H&E); **(B)** peripheral nerve (*) with perineural PDL-1 positive TC infiltration (left) and surrounding PDL-1 negative ICs (right); **(C)** high grade urothelial carcinoma with a dense intratumoral infiltration composed of PD-L1 and **(D)** CD68 positive macrophages; **(E)** muscle invasion with few PD-L1 positive tumor cells accompanied by PD-L1 positive mast cells (*); **(F)** PD-L1 immunohistochemistry as highlighted by a GIEMSA stain showing mast cells with granular purple violet cytoplasm.

In primary tumors expression of PD-L1 in TCs was patchy within the individual tumor and was observed in the invasive tumor component dominantly at the invasion front (Figure 1). ICs expressing PD-L1 were mostly detected surrounding invasive tumor cell nests, but few tumors also showed an intense intratumoral infiltration by PD-L1 positive ICs (Figure 2).

**Figure 3:**
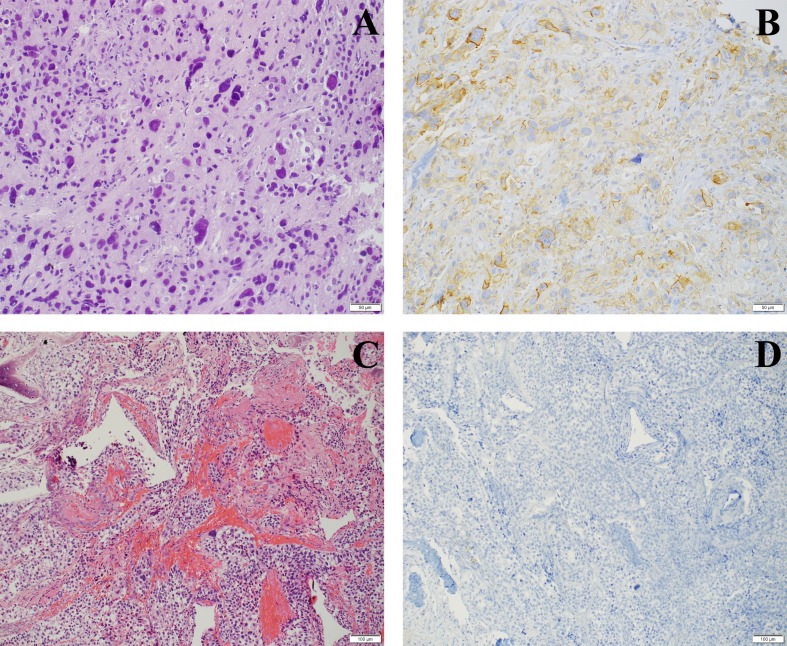
PD-L1 staining on TCs in metastatic sites **(A)** H&E staining of lung metastasis of a high grade urothelial carcinoma, **(B)** with high score of PD-L1 (IC2/3) expression; **(C)** bone metastasis (H&E), **(D)** being completely negative for PD-L1 (IC0).

The overall prevalence of PD-L1 IC0, IC1 and IC2/3 on TCs in RC tumor samples was 55.7% (n=34/61), 18.1% (n=11/61) and 26.2% (n=16/61). PD-L1 IC0, IC1 and IC2/3 expression on TCs in metastatic tumor samples was 63% (n=17/27), 18.5% (n=5/27) and 18.5% (n=5/27), including a representative example for IC0 and IC2/3 PD-L1 expression shown in Figure 3.

We confirmed a tendency towards a positive correlation between PD-L1 positivity on TCs and ICs, in primary tumors (r_s_= 0.267; *p*=0.038) as well as in metastasis (r_s_= 0.234; p=0.240), with a steadily increasing rate of PD-L1 positivity on ICs from IC0 to IC2/3 PD-L1 expression on TCs, Table 2. In primary tumors, PD-L1 negativity was shown in 55.7% (TCs) and 22.9% (ICs). Concerning biopsies of metastatic sites (n=27), PD-L1 negative expression was confirmed in 17 (62.9%) of 27 patients on TCs, and in 12 (44.4%) of 27 patients on ICs.

Table 2Bar charts of the association between PD-L1 expression (IC0, IC1, IC2/3) on tumor cells (TCs) and PD-L1 expression (negative, positive) on immune cells (ICs)

**Table:** A) Primary tumors (n=61)

	IC0	IC1	IC2/3
positive	67.6%	81.8%	93.8%
negative	32.4%	18.2%	6.2%
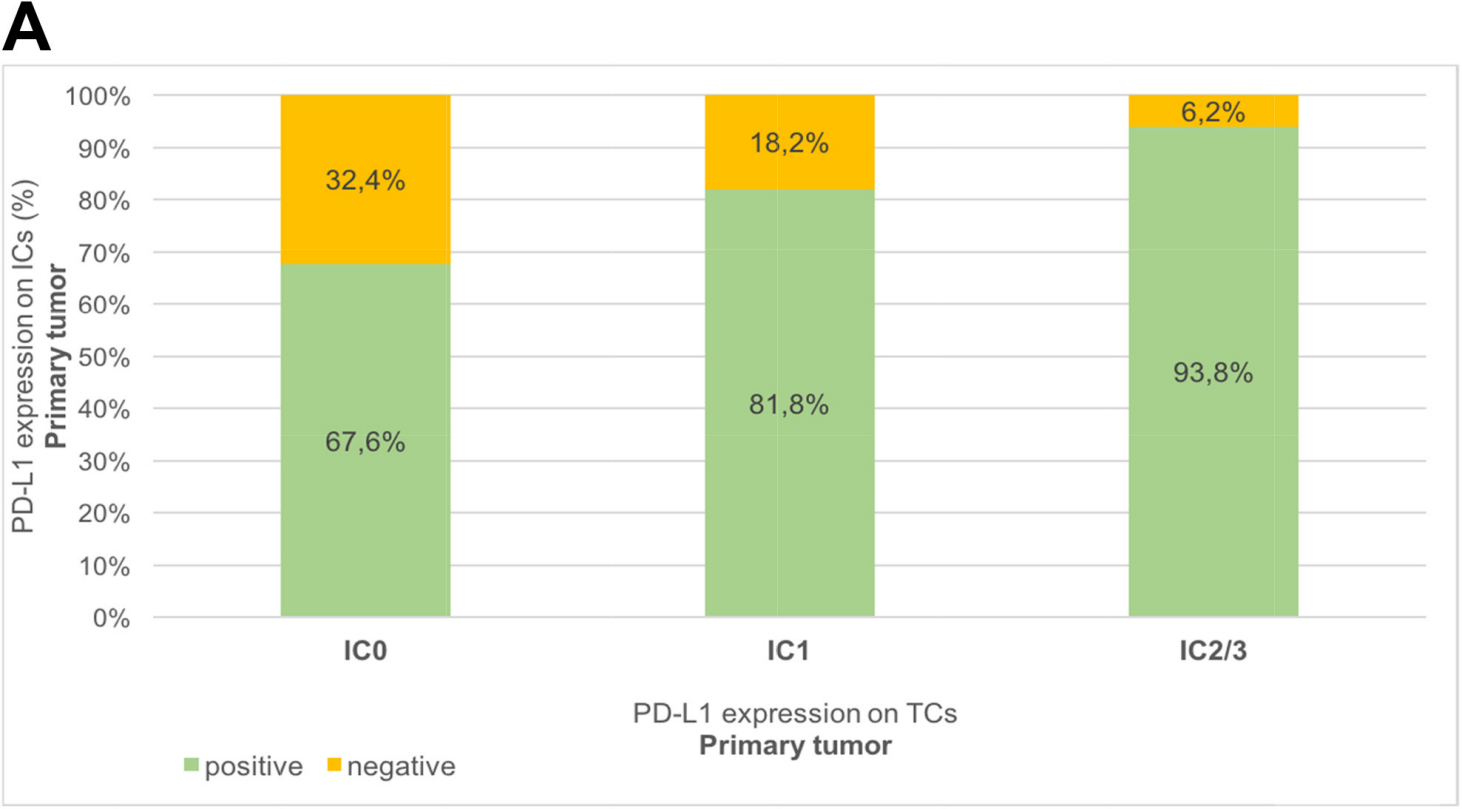

**Table:** B) Metastatic sites (n=27)

	IC0	IC1	IC2/3
positive	35.3%	60.0%	60.0%
negative	64.7%	40.0%	40.0%
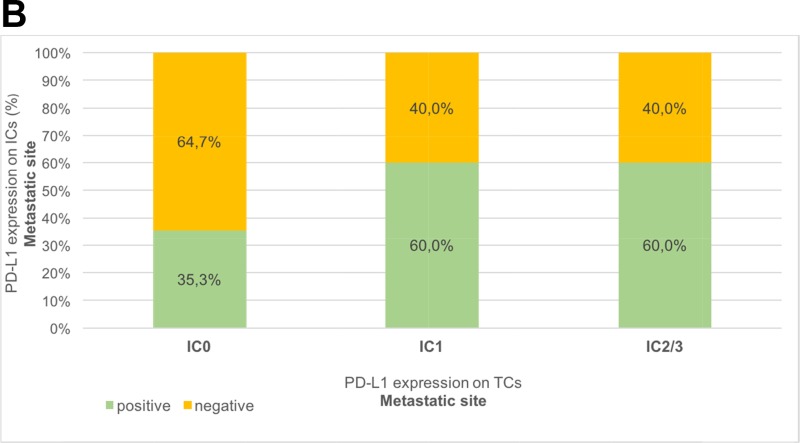			

On 27 paired samples, we evaluated dynamic changes of PD-L1 expression between primary tumor and metastasis, the PD-L1 score on TCs of the primary tumor correlated significantly with the PD-L1 score of the metastasis (r_s_= 0.520; *p*=0.005) with statistical homogeneity and equal distribution (*p*=0.637), confirming a low discordance rate of 22.2% (6/27 patients) with a PD-L1 positivity (IC1/2/3) on TCs of 59.3% in both primary tumors and metastases. Regarding PD-L1 expression on ICs, no significant correlation between primary tumors and metastases were shown (r_s_= 0.235; *p*= 0.239) with a high discordance rate of 44.4% (PD-L1 positivity tumor *vs*. metastasis: 77.7% *vs*. 44.4%), resulting in significant dynamic changes between primary tumor and metastasis (*p*=0.006).

### Association of PD-L1 expression and clinico-pathologic features

Table 3 shows the association between PD-L1 expression on TCs and ICs in RC specimens and clinico-pathologic features. High PD-L1 expression (IC2/3) on TCs was more frequently seen in patients with histologic subtype of urothelial cancer compared to patients with pure urothelial cancer (46.2% *vs*. 20.8%; *p*=0.002). There was no association between PD-L1 expression on TCs and ICs and any remaining histopathologic parameter, Table 3.

**Table 3:** Association of PD-L1 expression and clinico-pathological characteristics

Parameters	PD-L1 expression on TCs			P-value*	PD-L1 expression on ICs		P-value*
	Primary tumor n (%)				Primary tumor n (%)		
	IC0	IC1	IC2/3		Negative	Positive	
**Age**				0.934			0.353
<65 (N=20)	12 (60.0%)	3 (15.0%)	5 (25.0%)		17 (85.0%)	3 (15.0%)	
>65 (N=41)	22 (53.7%)	8 (19.5%)	11 (26.8%)		30 (73.2%)	11 (26.8%)	
**Gender**				0.757			0.713
Male (N=48)	28 (58.3%)	8 (16.7%)	12 (25.0%)		36 (75.0%)	12 (25.0%)	
Female (N=13)	6 (46.1%)	3 (23.1%)	4 (30.8%)		11 (84.6%)	2 (15.4%)	
**Tumor stage**				0.580			0.382
≤pT2b (N=22)	14 (63.6%)	4 (18.2%)	4 (18.2%)		16 (72.7%)	6 (27.3%)	
≥pT3a (N=39)	20 (51.3%)	7 (17.9%)	12 (30.8%)		31 (79.5%)	8 (20.5%)	
**Concomitant CIS**				0.885			0.126
No (N=27)	14 (51.9%)	5 (18.5%)	8 (29.6%)		18 (66.7%)	9 (33.3%)	
Yes (N=34)	20 (58.8%)	6 (17.7%)	8 (23.5%)		29 (85.3%)	5 (14.7%)	
**R Positivity**				0.845			0.277
No (N=47)	27 (57.4%)	8 (17.1%)	12 (25.5%)		38 (80.9%)	9 (19.1%)	
Yes (N=14)	7 (50.0%)	3 (21.4%)	4 (28.6%)		9 (64.3%)	5 (35.7%)	
**LVI**				0.436			0.331
No (N=23)	11 (47.8%)	6 (26.1%)	6 (26.1%)		17 (73.9%)	6 (26.1%)	
Yes (N=33)	20 (60.6%)	4 (12.1%)	9 (27.3%)		28 (84.8%)	5 (15.2%)	
**Number of resected LNs**				0.940			0.553
≤15 (N=26)	15 (57.7%)	4 (15.4%)	7 (26.9%)		19 (73.1%)	7 (26.9%)	
>15 (N=35)	19 (54.3%)	7 (20.0%)	9 (25.7%)		28 (80.0%)	7 (20.0%)	
**LN positivity**				0.873			0.527
No (N=40)	22 (55.0%)	8 (20.0%)	10 (25.0%)		32 (80.0%)	8 (20.0%)	
Yes (N=21)	12 (57.1%)	3 (14.3%)	6 (28.6%)		15 (71.4%)	6 (28.6%)	
**Histologic subtype**				**0.002**			1.000
No (N=48)	32 (66.7%)	6 (12.5%)	10 (20.8%)		37 (77.1%)	11 (22.9%)	
Yes (N=13)	2 (15.4%)	5 (38.5%)	6 (46.1%)		10 (76.9%)	3 (23.1%)	

CIS=carcinoma *in situ*; LVI=lymphovascular invasion; LN=lymph node; P values by Fisher’s exact test; *p<0.05; **p<0.01; ***p<0.001.

*From Fisher’s exact test.

### Recurrence-free survival (RFS) from RC to local or distant metastatic spread

**Figure 4:**
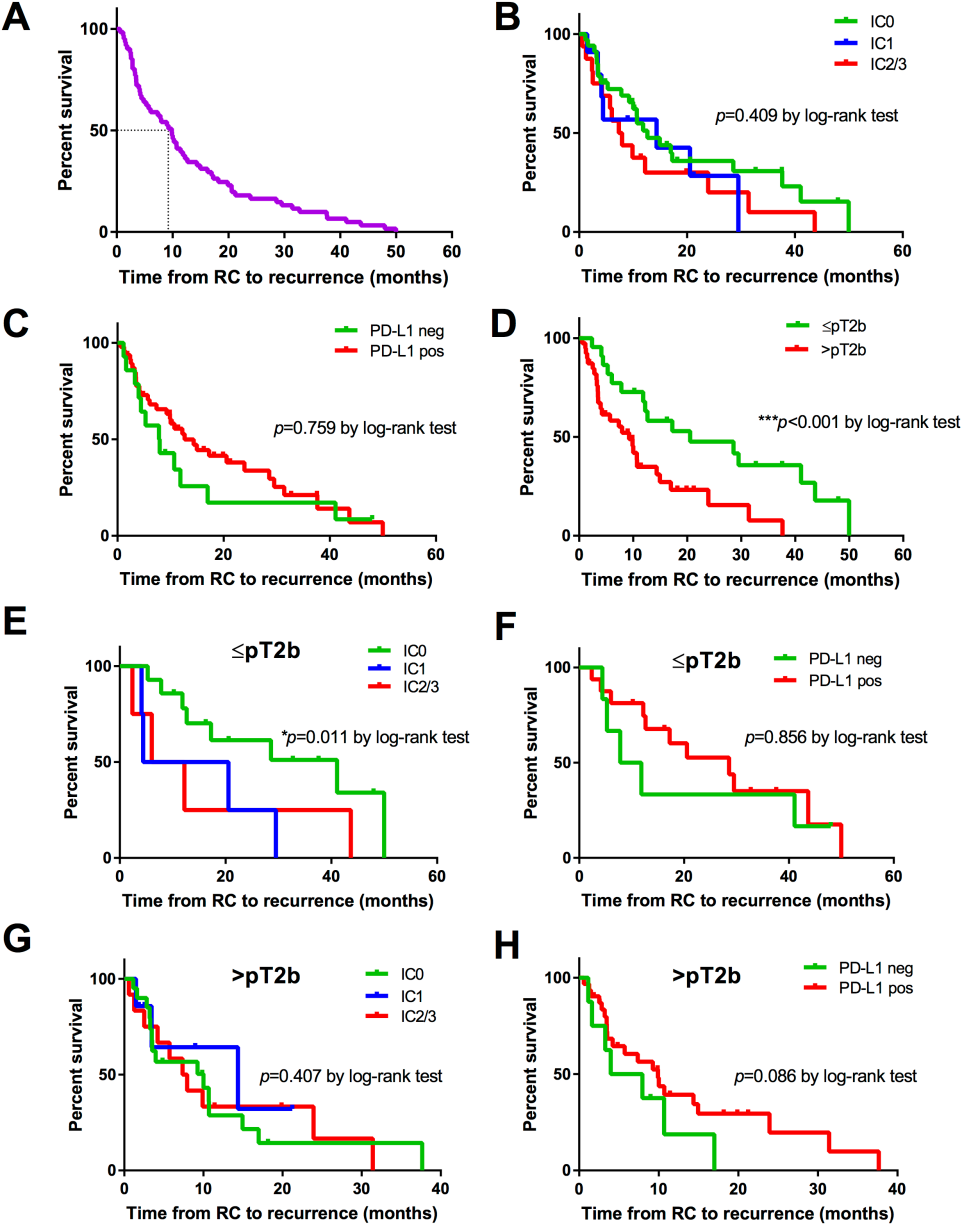
Kaplan-Meier survival curves for recurrence-free survival (RFS) in months **(A)** Overall RFS in all patients (n=61). **(B)** RFS according to PD-L1 score (IC0: n=34, IC1: n=11, IC2/3: n=16) on TCs of primary tumor. **(C)** RFS according to positive (n=47) or negative (n=14) PD-L1 staining on TILs of primary tumor. **(D)** RFS based on pathological tumor stage (≤pT2b: n=22 *vs*. ≥pT3a; n=39). **(E** and **F)** RFS according to PD-L1 score (IC0: n=14, IC1: n=4, IC2/3: n=4) on TCs and PD-L1 staining (positive: n=16, negative: n=6) on TILs of primary ≤pT2b tumors. **(G** and **H)** RFS according to PD-L1 score (IC0: n=20, IC1: n=7, IC2/3: n=12) on TCs and PD-L1 staining (positive: n=31, negative: n=8) on TILs of primary ≥pT3a tumors. *P* values by log-rank test; *p<0.05; **p<0.01; ***p<0.001.

The median recurrence-free survival from RC was 9.8 months (95% CI 7.2-12.5) in all patients (Figure 4A) and was similar between the different PD-L1 tumor cell expression (IC0: median [95% CI] RFS 10.2 months [8.9-11.4]; IC1: 4.4 [0.0-10.3] and IC2/3: 7.3 [3.6-11.1 months]; *p*=0.409) and immune cell groups (positive: median RFS 10.0 months [8.1-11.9] and negative: 7.8 [2.9-12.7]; *p*=0.759) without statistically significant differences, Figure 4B-4C.

Extra-vesical tumor stage [HR=2.76; 95% CI: 1.54-4.92; *p*=0.001] was the sole histopathological parameter being associated with poor RFS (≤pT2: median RFS 16.3 months [6.7-25.8] and ≥pT3: 5.7 [0.8-10.6]; *p*<0.001), respectively (Figure 4D). Stratifying tumor cell and immune cell PD-L1 groups by tumor stage at RC, patients with an increased PD-L1 score on TCs (IC2/3 *vs*. IC0: median RFS 6.1 *vs*. 17.2 months for ≤pT2 tumors; *p*=0.362; and 7.4 *vs*. 3.9 months for ≥pT3 tumors; *p*=0.760) and a negative PD-L1 score on ICs (negative *vs*. positive: median RFS 7.8 *vs*. 17.2 months for ≤pT2 tumors; *p*=0.854; and 3.9 vs. 5.7 months for ≥pT3 tumors; *p*=0.324) showed a tendency towards shorter RFS, Figure 4E-4H.

### Disease-specific survival (DSS) from recurrence

**Figure 5:**
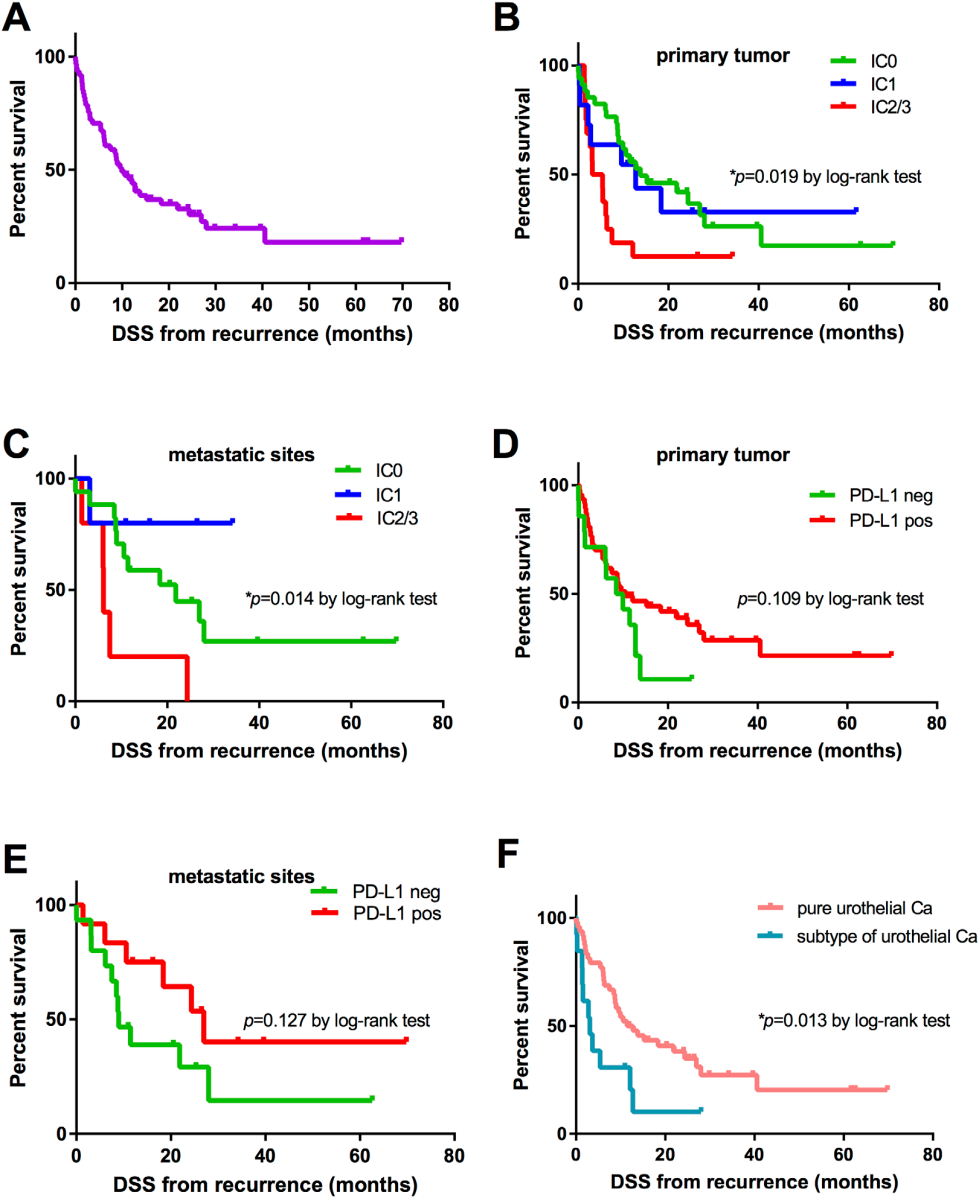
Kaplan-Meier survival curves for disease-specific survival (DSS) in months **(A)** Overall DSS in all patients (n=61). **(B** and **C)** DSS according to PD-L1 score (IC0: n=34, IC1: n=11, IC2/3: n=16) on TCs of primary tumor and metastatic site (IC0: n=17, IC1: n=5, IC2/3: n=5). **(D** and **E)** DSS stratified by PD-L1 expression (positive *vs*. negative) on TILs of primary tumor (positive: n=47, negative: n=14) and metastasis (positive: n=12, negative: n=15). **(F)** DSS based on histologic subtypes of urothelial cancer (pure urothelial cancer: n=48 vs. variants of urothelial cancer: n=13). *P* values by log-rank test; *p<0.05; **p<0.01; ***p<0.001.

With a median DSS from recurrence of 9.9 months (95% CI 6.2-13.7) in all patients (Figure 5A), patients in the IC2/3 group from primary tumor (median [95% CI] CSS 3.2 months [0.0-7.7]) and from metastatic site (median [95% CI] CSS 6.1 months [5.9-6.3]) showed a statistically significant poorer DSS compared to patients in the IC0 group (primary tumor: median [95% CI] CSS 13.8 months [1.1-26.5], *p*=0.019; metastatic site: median [95% CI] CSS 21.8 months [3.5-40.2], *p*=0.014) despite platinum-based chemotherapy, Figure 5B-5C. The 6-month DSS rate was 82.4% in the IC0 group, 63.6% in the IC1 group and 37.5% in the IC2/3 group on TCs of primary tumor, and 88.2% (IC0), 80% (IC1) and 80% (IC2/3) concerning the PD-L1 score on TCs of metastatic sites.

The PD-L1 expression on ICs of primary tumors and metastatic sites had no significant influence on DSS (median DSS, positive *vs*. negative: 10.5 *vs*. 8.5 months [primary tumor], *p*=0.109; 26.9 *vs*. 8.9 months [metastatic site], *p*=0.127), Figure 5D-5E.

Interestingly, patients with histologic variants of urothelial cancer (median [95% CI] DSS 3.1 [0.6-5.6] months) responded less to platinum-based chemotherapy with a shorter DSS than those patients with pure urothelial cancer (median [95% CI] DSS 11.4 [5.3-17.6] months, *p*=0.013), Figure 5F. Univariate Cox regression analysis revealed that histological variants of urothelial cancers [HR = 2.35; 95% CI: 1.17-4.71; *p*=0.016], and a high IC2/3 PD-L1 expression on TCs of primary tumor [HR=2.53; 95% CI: 1.27-5.03; *p*=0.008] as well as of metastatic sites [HR=3.55; 95% CI: 1.15-10.89; *p*=0.027] were associated with a higher risk of poor DSS after recurrence. Moreover, a high PD-L1 expression on TCs was associated with histologic subtypes of urothelial cancer (*p*=0.002).

### Overall survival (OS) from RC

**Figure 6:**
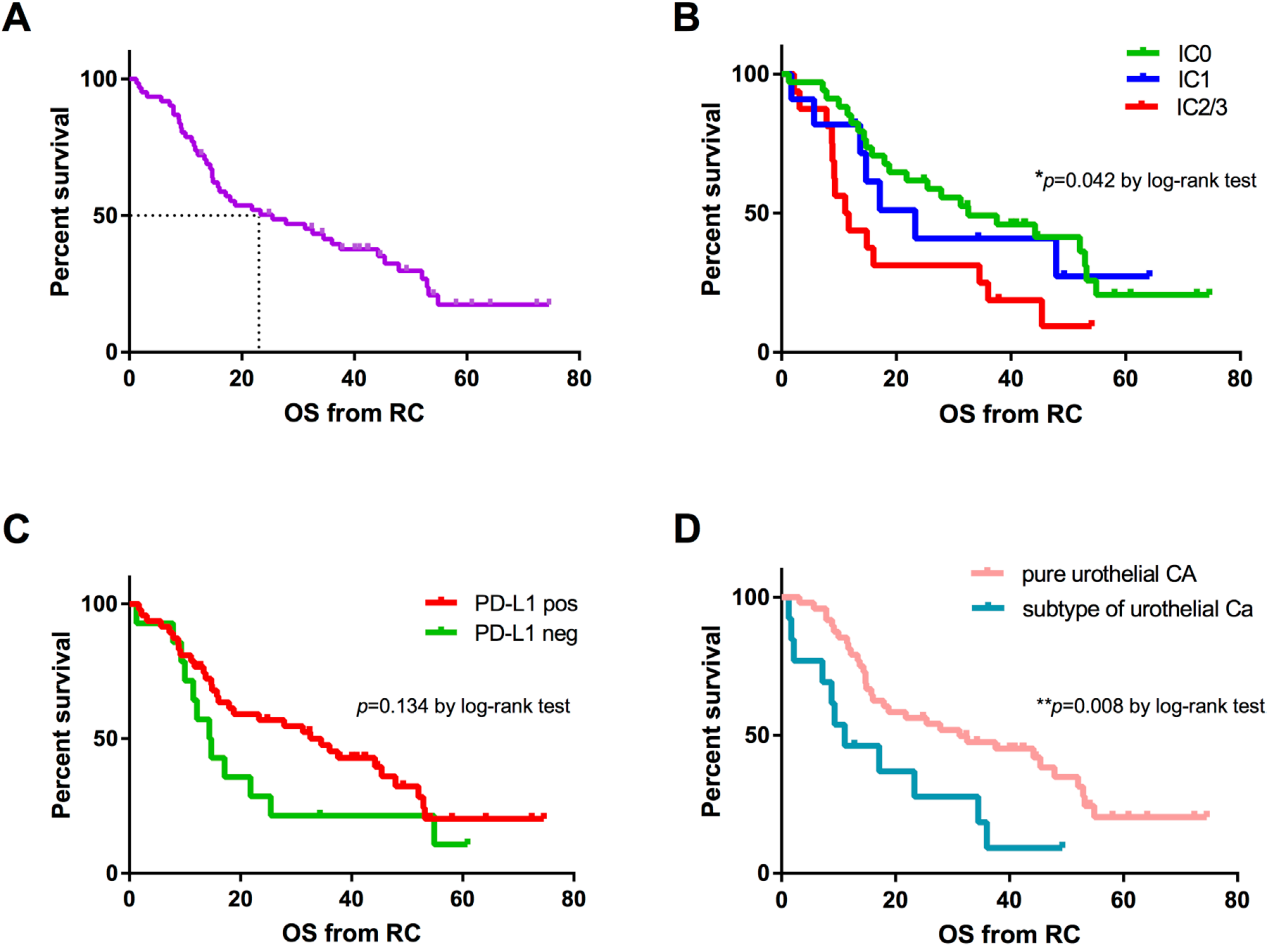
Kaplan-Meier survival curves for overall survival (OS) in months **(A)** Median OS in all patients (n=61). **(B)** OS according to PD-L1 score (IC0: n=34, IC1: n=11, IC2/3: n=16) on TCs of primary tumor. **(C)** OS based on PD-L1 staining (positive: n=12 vs. negative: n=15) on TILs of metastatic sites. **(D)** OS stratified by histologic subtypes of urothelial cancer (n=13) versus pure urothelial cancer (n=48) at RC specimens. *P* values by log-rank test; *p<0.05; **p<0.01; ***p<0.001.

The median OS after RC was confirmed with 25.5 (95% CI 10.1-40.8) months in all patients, Figure 6A. The PD-L1 score on TCs from RC specimens significantly influenced the OS after RC. Patients with a IC0 score (median [95% CI] OS 32.6 [12.^9^–^52^.^1^] months) had a significant better OS than patients with a IC1 score (median [95% CI] OS 23.3 [10.5-36.2] months) or IC2/3 score (median [95% CI] OS 11.1 [6.4-15.8] months; *p*=0.042), Figure 6B. Nevertheless, a positive PD-L1 expression on TILs had no influence on OS (high *vs*. low PD-L1 score: median [95% CI] OS 32.6 [17.9-47.2] *vs*. 14.4 [9.7-19.1] months; *p*=0.134), Figure 6C.

Focusing on histopathological factors, patients with histological subtypes of urothelial carcinoma (median OS: 11.1 months) showed to have a significant poorer OS than patients with pure urothelial carcinoma in the RC specimens (median OS: 31.2 months; *p*=0.008), Figure 6D. Univariate analysis confirmed a significant positive association between IC2/3 score on TCs of primary tumor [HR=2.25; 95% CI: 1.15-4.41; *p*=0.019], histological variants of urothelial cancer [HR=2.48; 95% CI: 1.23-5.01; *p*=0.011] and lower OS.

## DISCUSSION

Despite radical cystectomy (RC) and pelvic LN dissection in localized MIBC, approximately 50% of patients will relapse on follow-up, depending on pathological tumor stage and LN status at RC specimens [19]. Cisplatin-based chemotherapy regimens such as gemcitabine/cisplatin (GC) or methotrexate, vinblastine, adriamycin and cisplatin (MVAC) are still the standard of care since the late 1980s [20], improving median survival from 3-6 months [21] to up to 14.0 and 15.2 months [22]. However, 50% of patients at metastatic disease are unfit for cisplatin-containing chemotherapies [23], with the necessity to use carboplatin-based regimes such as carboplatin/gemcitabine or methotrexate, carboplatin, vinblastine (M-CAVI), and thus resulting in lower survival rates (median OS: 9.3 months for gem/carbo and 8.1 months for M-CAVI), [24]. Therefore, novel treatment options besides traditional modalities as surgery and chemotherapy are urgently needed, especially for patients with limited options of durable therapeutic response [25].

The high accessibility of bladder cancer to immunotherapy is well known since the introduction of BCG in 1976 [26], still belonging to the standard adjuvant treatment in high-risk NMIBC. Intravesical BCG instillations activate the innate and adaptive immune system leading to bladder cancer rejection via increased infiltration and activation of TILs. However, not all patients benefit from BCG therapy or develop evasive immune escape mechanisms, which include induction of TILs exhaustion via PD-L1 overexpression or induction of immunosuppressive molecules [27]. Mechanistically high PD-L1 expression suggests tumor-associated immune tolerance and escape from immune surveillance, which is one of the hallmarks of cancer cell immune evasion [28]. Thereby, immune checkpoints (ICPs) play an important role in cancer cell immune evasion and are expressed by tumors to escape T-cell mediated lysis. Recently, many ICPs molecules have been described and targeted for activating the immune system and unleash anti-tumor immunity to eliminate bladder cancer cells, Supplementary Figure 1. The most important ICP targets are the cytotoxic T-lymphocyte antigen 4 (CTLA-4) and the programmed death 1 (PD-1) receptor and its major ligand PD-L1 [29]. Inhibition of CTLA-4 proved to induce long-term responses, however more frequent immune related adverse events were reported compared to the inhibition of PD-1/PD-L1 axis [30]. The first two FDA-approved ICP inhibitors for bladder cancer are atezolizumab [9] and nivolumab [28]. Avelumab another PD-L1 ICP inhibitor is under clinical testing (NCT02603432) [31], and the two ICP inhibitors durvalumab [2] and pembrolizumab [32] showed significant prolonged OS in metastatic urothelial carcinoma patients that progressed after platinum-based chemotherapy, thus being currently in the approval phase. An overview of ongoing immunotherapy trials in bladder cancer is shown in Supplementary Figure 2.

Prognostic significance of PD-L1 protein expression has been studied in many tumor entities [33] and its relevance as predictive biomarker for treatment stratification for ICP inhibitors is still under critical evaluation. Furthermore, the role of PD-L1 as efficient predictive factor in metastatic bladder cancer for selecting patients who benefit most from immunotherapy is controversially discussed [12, 16–18]. The predictive value of PD-L1 is limited due to a lack of uniform definition of PD-L1 positivity, a lack of standard assays for PD-L1 expression [18], with different semi-quantitative scores of the PD-L1 expression status in clinical trials, different PD-L1 antibody clones, and thus resulting in heterogeneous data [2-3, 9, 28, 32-33]. Therefore, standardized criteria for PD-L1 positivity are urgently needed [34]. Moreover, tumor heterogeneity can be a reason that PD-L1 expression status may differ between primary tumor and metastatic lesions, hypothesizing that the predictive accuracy of PD-L1 is depending on the time of biopsy, being related to previous therapies [18, 33].

PD-L1 expression can occur on the TCs or on TILs [17]. Thus, we evaluated PD-L1 in both TCs and ICs. We show a tendency towards a positive correlation of PD-L1 positivity between TCs and ICs, in primary tumor as well as in metastasis.

Moreover, PD-1 expression correlated between primary tumors and metastatic lymph nodes with a concordance rate of 78% [35], without dynamic changes in PD-1 expression before and after neoadjuvant chemotherapy in bladder cancer [16]. In our study, consistent PD-L1 positivity on TCs was noticed between primary tumor and metastatic site, suggesting a low relevance in the timing of tissue sample retrieval when staining PD-L1 on TCs [16]. This fact may be important in clinical study designs testing efficacy of ICP inhibitors where patients are often stratified by a PD-L1 expression score [2, 9, 28, 32]. The correct timing of obtaining tissue samples for PD-L1 staining (primary tumor, before any kind of chemotherapy) and staining localization (TCs versus ICs) remains an issue of debate [16].

In addition to PD-L1, other potential biomarkers including state of the art molecular subtyping, in depth TCR sequencing, somatic mutational density quantification, and identification of T cell-inflamed/non-T cell-inflamed tumor microenvironments using immune gene expression profiling are currently under investigation with promising results [7, 36], highlighting the complexity and dynamic changes of the antitumor immune response [13], with the necessity for combined biomarkers. Nevertheless, preliminary data suggested a significant relationship between PD-L1 expression, clinical outcome, radiation response and therapeutic response to immunotherapy in bladder cancer [2, 9, 28, 37].

Here, we describe that higher PD-L1 expression on TCs in chemotherapy-naive RC specimens is correlated with poor clinical outcome in terms of DSS and OS in patients who developed metastatic disease and were homogeneously treated with first line platinum-based chemotherapy. In line with our results on the prognostic role of PD-L1 positivity on TCs it has been reported that PD-L1 expression was associated with a shorter survival after RC [35, 38–39], whereas other trials showed no influence on RFS and OS [40]. Furthermore, we can show that PD-L1 may be an efficient predictive biomarker in metastatic bladder cancer patients for chemotherapy resistance, as already described by Zhang *et al* (2016) in non-small-cell lung cancer patients undergoing cisplatin-based neoadjuvant chemotherapy [41]. Moreover, knockdown of PD-L1 expression increased chemo-response to cisplatin *in vivo* and *in vitro* [41]. These results are underlined in the clinical setting by the KEYNOTE-024 study in lung cancer showing therapeutic superiority of pembrolizumab over platinum-based chemotherapy with increased PFS and OS in patients with a high PD-L1 tumor proportion score ≥50% [42]. In mammary epithelial cells, MERTK overexpression a member of the TAM (TYRO3, AXL, and MERTK) receptor tyrosine kinases, promotes chemo-resistance and induces consecutive PD-L1 overexpression [43]. Vice versa MERTK knockdown significantly reduced PD-L1 expression levels in highly invasive breast cancer cells MDA-MB 231 [43]. In bladder cancer, FGF2 was significantly increased in chemo-resistant bladder cell lines, stimulating endothelial cell migration, growth and tube formation by producing FGF2 [44], and thus playing an important role in the development of a cisplatin-resistant phenotype [45]. A possible explanation for poor prognosis of FGF-2 expressing bladder cancers is the association with epithelial to mesenchymal transition (EMT), high proliferation, low mutation load and high expression of CTLA-4, PD-1 and PD-L1, thus being more sensitive to immune checkpoint inhibition [46]. In line with these *in-vitro* findings, we are able to show that those patients with histological subtypes of urothelial cancer (including approximately 70% with squamous differentiation, in which FGFR alterations are well known [47]) had significant increased PD-L1 expression, but decreased survival outcomes compared to pure urothelial cancers. Nevertheless, results in bladder cancer are inconsistent. Erlmeier *et al* (2016) confirmed no significant association between PD-L1 status and response to neoadjuvant or adjuvant chemotherapy in MIBC [16]. Baras *et al* (2016) [8] also showed that PD-L1 expression on TCs was not a significant predictor of response to neoadjuvant chemotherapy [8]. Concerning molecular subtypes of MIBC, p53-like [48] and claudin-low tumors [15] were identified as being consistently resistant to neoadjuvant chemotherapy. Claudin-low MIBCs were uniformly enriched for immune gene signatures in addition to immune checkpoint molecules, showing that these tumors are immune infiltrated and simultaneously actively immunosuppressed, with low PPAR-g activity, high NFKB activity, inducing pro-inflammatory milieu being linked with immunotherapy response [15]. In contrast, basal tumors are highly aggressive tumors benefiting mostly from neoadjuvant chemotherapy (3-yr OS rate: 77.8% *vs*. 49.2%; p<0.001), [14]. These chemo-sensitive subtypes of MIBC confirmed an increased p63 gene signature, being associated with active PPAR-g as also reported for ovarian cancer [49]. These findings may support the hypothesis that the tumor microenvironment and immune system influence response to immunotherapy and chemotherapy [8], meaning that immune-infiltrated and actively immunosuppressed tumors are more suitable for immunotherapy, whereas non-T cell-inflamed tumors respond more to chemotherapy [7, 41–42].

Our results must be interpreted with cautions, as several limitations exist: we included a relatively small sized patient cohort from a single-center institution with retrospective and observational study design. Concerning statistical limitations, there is lack of multivariate analyses due to multicollinearity, moreover, excluding those patients who developed recurrence and were followed-up elsewhere postoperatively may cause a selection bias. However, our findings of this study are clearly hypothesis generating, identifying a pathophysiological link between PD-L1 expression and therapy response. These preliminary findings are claiming for further validation in prospective, multicenter trials as well as for experimental studies analyzing gene expression profiling of tumors as well as of tumor microenvironment to get more insights how the immune phenotype can influence response to immunotherapy and chemotherapy.

## MATERIALS AND METHODS

### Patients

This study was approved by the local ethics committee of the Medical University Innsbruck (study number 1006/2017) and was performed according to the principles of the Declaration of Helsinki and its subsequent amendments [50]. Reviewing medical records from MIBC patients who underwent radical cystectomy with extended pelvic lymph node dissection and consecutive oncologic follow-up at our department, a series of 61 patients who developed local recurrence or distant metastasis after RC followed by platinum-based chemotherapy at the time of recurrence as first-line regime was included in the study. Descriptive and histopathological data of patients were assessed using our cystectomy database. All included patients received neither neoadjuvant nor adjuvant platinum-based chemotherapy. Follow-up intervals and detailed clinical investigations on surveillance after RC according to our institutional practice have been published recently [51]. Tumor progression was determined by standard imaging, and histologically confirmed by CT guided biopsy (due to uncertain diagnosis of metastatic lesions) in 27 patients prior to starting first-line chemotherapy

### Tumor samples and regions

All 61 cystectomy specimens were reviewed for diagnosis, tumor grade (according to the WHO 1973 and 2004) and tumor stage (according to the actual TNM) by two experienced uropathologists (BA and SS). Further pathology parameters such as the presence of lymphovascular invasion (LVI), surgical resection margins (R status), pelvic lymph node (LN) infiltration, total number of resected LNs, number of positive LNs, and variants of urothelial carcinoma (micropapillary, sarcomatoid, squamous differentiation, adeno variant, nested) have routinely been reported. Of every case one to three representative blocks were selected for further analysis including superficial tumor, if available, invasive tumor and invasion front.

We further compared the dynamic changes in PD-L1 expression of primary tumors and metastasis, and the corresponding chemotherapy response (DSS) and outcome. We assessed PD-L1 expression in 27 patients with matched biopsy tissues from metastasis (resulting in 29 tumor specimens: one patient had metastasis in lung and liver, one had a local recurrence and lung metastasis) and RC samples.

### Immunohistochemistry (IHC)

For detection of PD-L1 expression a monoclonal antibody against PD-L1 (Clone CLA-10, Biocare, United Kingdom), which has been validated at the Division of Pathology, Medical University of Innsbruck, was used. Human placental tissue served as positive control.

Staining was performed using an automated immunostainer (BenchMark ULTRA, Ventana Medical Systems, Tucson, US). Briefly, formalin-fixed, paraffin-embedded (FFPE) tissue sections were cut in widths of 1.5 μM. After deparaffinization, the slides were treated with cell conditioning reagent 1 (CC1, Ventana Medical Systems, Tucson, US) for antigen retrieval. Primary antibodies were incubated for 32 minutes at 37°C. The Ultra View DAB Detection Kit (Ventana Medical Systems, Tucson, US) was used for visualization, in accordance with the manufacture’s recommendation. Finally, slides were washed in distilled water, counterstained with hematoxylin (12 minutes) and bluing reagent (4 minutes), dehydrated in a descending order of alcohols, cleared in xylene, and coverslipped with Tissue-tek mounting medium (Medite, Germany).

### Quantification of PD-L1 on tumor cells (TCs) and tumor-infiltrating immune cells (ICs) of primary tumor and metastatic site

Analysis of PD-L1 staining was done separately for expression on TCs as well as on tumor-infiltrating ICs. Expression on TCs was evaluated as positive or negative and the exact percentage (%) of positive TCs was noted; in addition, a semi-quantitative score for the PD-L1 expression status on TCs in the tumor microenvironment was applied for statistical analysis and defined by the percentage of PD-L1-positive TCs as published previously [9]: < 1%= Score 0 (IC0), ≥1% but <5% = Score 1 (IC1), ≥5 % = Score 2/3 (IC2/3) in primary tumors (n=61) as well as at the metastatic site (n=27).

Inflammation was scored as weak, intermediate and strong and the composition of the inflammatory infiltrate was described (lymphocytes, plasma cells, eosinophilic granulocytes) [52]. PD-L1 expression on ICs was evaluated as either present (positive) or absent (negative) for both the primary tumor and the metastatic site.

### Statistics

All patient-, clinico- and histopathologic characteristics as well as PD-L1 expression levels were analyzed descriptively by giving frequencies or means, standard deviations, and medians, as appropriate. Categorical variables were compared in bivariate analyses using Fisher’s exact test. Correlations between various PD-L1 expression levels were assessed using Spearman’s ρ correlation coefficient (r_s_). Dynamic changes in PD-L1 expression levels between primary tumor and metastatic sites were analyzed in matched pair analyses with marginal homogeneity test and McNemar test, separately for expression levels in tumor cells and immune cells. Recurrence-free survival (RFS), disease-specific survival (DSS), and overall survival (OS) were investigated through Kaplan-Meier survival analysis, survival curves were plotted, and the influence of various factors on survival was tested using log-rank tests. For all statistical tests, a significance level of α=0.05 (two-tailed) was applied. SPSS, version 22.0 (IBM Corp., Armonk, NY, USA) was used for statistical analysis.

## CONCLUSION

Bladder cancer is a heterogeneous tumor with divergent PD-L1 expression levels between TCs and TILs, in primary tumors and metastases. Analyzing a matched group, significant dynamic changes of PD-L1 positivity on ICs were shown in RC specimens (77.7%) and subsequent metastasis (44.4%). The most stable parameter was the PD-L1 score on TCs, with homogeneous PD-L1 positivity in both primary tumors and metastatic sites. This important pathological finding suggests that PD-L1 can either be determined in the primary tissue as well as in metastasis and implicates similar biological behavior of primary and metastatic tumor. High PD-L1 expression was associated with histologic subtypes of urothelial cancers and had a negative predictive value for response to first-line chemotherapy. Furthermore, high PD-L1 expression on TCs was also a negative prognostic marker for DSS and OS. Thus, PD-L1 may be a potential target in predicting response to chemotherapy in metastatic bladder cancer, being a possible promising strategy for individualized targeted therapy with selection of patients that mostly benefit from chemotherapy or immunotherapy. Further trials are necessary to prove these preliminary data and to elaborate mechanistic aspects of chemo-resistance and PD-L1 overexpression in bladder cancer.

## SUPPLEMENTARY MATERIALS FIGURES


