# Supplementary material for: PD-L1 expression in bladder cancer and metastasis and its influence on oncologic outcome after cystectomy
Source: Oncotarget. 2017 Aug 3;8(40):66849–64. doi: 10.18632/oncotarget.19913 (PMC5620140; doi:10.18632/oncotarget.19913)
Supplement: Supplementary file 1 [file oncotarget-08-66849-s001.pdf]

PD-L1 expression in bladder cancer and metastasis and its influence on oncologic outcome after cystectomy

SUPPLEMENTARY MATERIALS

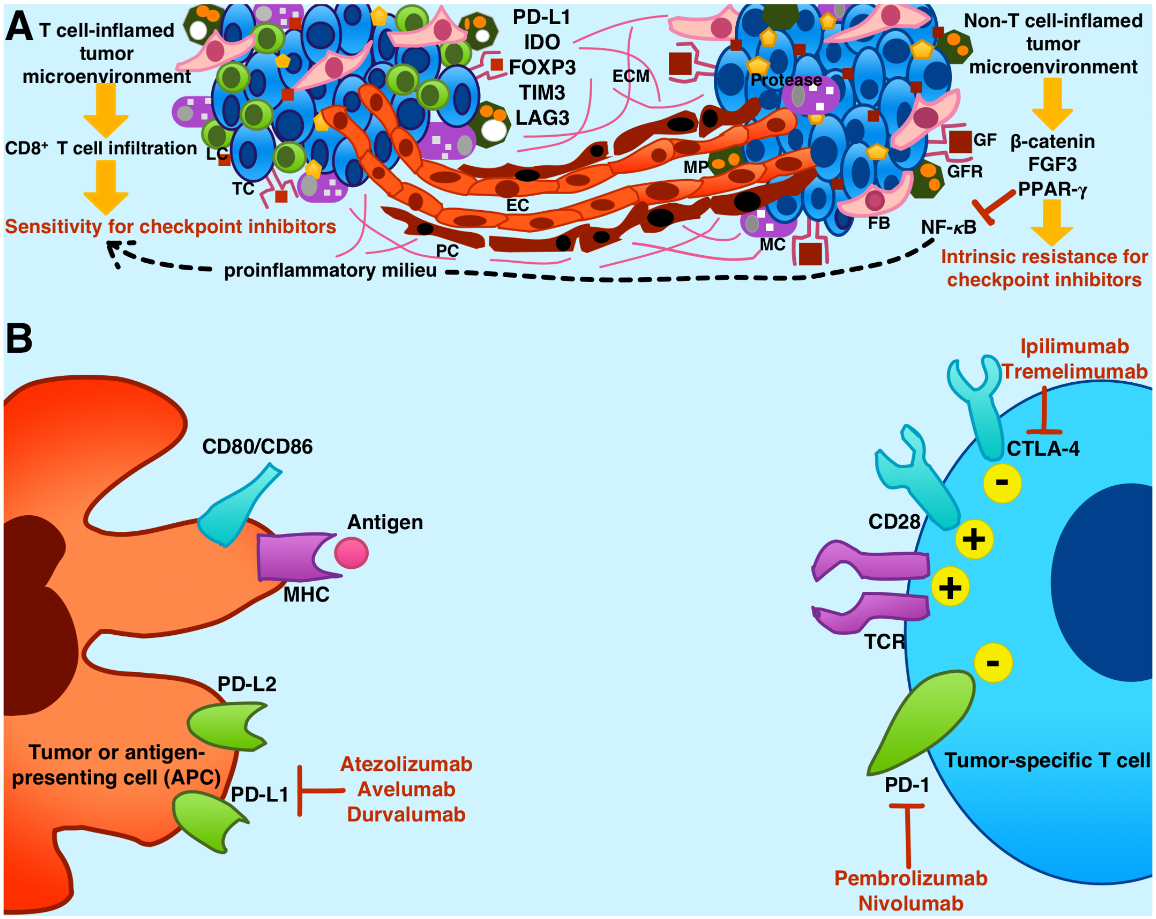

Supplementary Figure 1: (A) The tumor microenvironment as a possible target in predicting response or intrinsic resistance to immunotherapy. The presence of intratumoral CD8+ T cells correlates with T cell-inflamed gene expression signature, and other immune-inhibitory markers such as IDO, FOXP3, TIM3 and LAG3. On the contrary, non-T cell-inflamed tumors have activated b-catenin, mutated FGF3 and PPAR-g, blocking NFKB and inhibiting a pro-inflammatory milieu, and thus may be responsible for intrinsic resistance to immunotherapy [7]. Abbreviations: EC=endothelial cell; ECM=extracellular matrix; FB=fibroblast; GF=growth factor; GFR=growth factor receptor; LC=lymphocyte; PC=pericyte; TC=tumor cell; MC=mast cell; MP=macrophage; **(B)** Mode of action of immune checkpoint (ICP) inhibitors currently tested in clinical trials of urothelial cancer. Two signals are necessary for proliferation, differentiation and activation of T cells: the T cell receptor (TCR) interacts with the antigen presented by MHC molecules. However, this step is insufficient for producing an effective T cell response by itself. Costimulatory signals binding between CD28 (T cell) and CD80/68 (antigen-presenting cell, APC) are required. CTLA-4 can bind to CD80/68 through a much higher affinity than CD28 resulting in a downregulation and functional inactivation of T cells. CTLA-4 inhibitors such as Ipilimumab and Tremelimumab can block this mechanism. PD-1 expression on T cells is induced after binding to its ligands, PD-L1 and PD-L2, which are upregulated on cancer cell surface of different cancer entities. PD-1 inhibits T cell activation. Pembrolizumab (MK3475) and Nivolumab are both PD-1 antibodies blocking the binding of both ligands, PD-L1 and PD-L2; Avelumab, Atezolizumab (MPDL3280A) and Durvalumab are specific PD-L1 monoclonal antibodies.

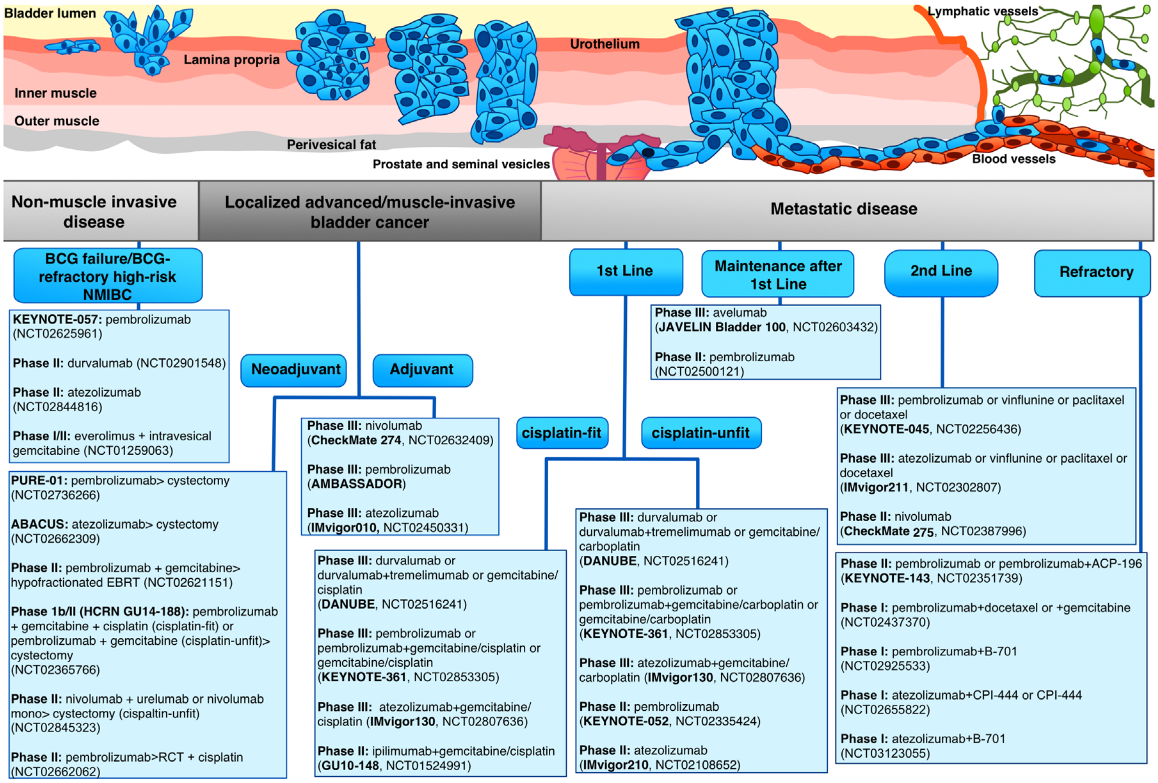

Supplementary Figure 2: Schematic summary of ongoing clinical trials evaluating therapeutic efficacy of immune checkpoint (ICP) inhibitors in different disease stages of bladder cancer: From BCG-resistant NMIBC, locally advanced, localized MIBC to metastatic disease (first-line, second-line, maintenance and refractory).
